# Evolution of an Eurasian Avian-like Influenza Virus in Naïve and Vaccinated Pigs

**DOI:** 10.1371/journal.ppat.1002730

**Published:** 2012-05-31

**Authors:** Pablo R. Murcia, Joseph Hughes, Patrizia Battista, Lucy Lloyd, Gregory J. Baillie, Ricardo H. Ramirez-Gonzalez, Doug Ormond, Karen Oliver, Debra Elton, Jennifer A. Mumford, Mario Caccamo, Paul Kellam, Bryan T. Grenfell, Edward C. Holmes, James L. N. Wood

**Affiliations:** 1 Cambridge Infectious Diseases Consortium, Department of Veterinary Medicine, University of Cambridge, Cambridge, United Kingdom; 2 Medical Research Council-University of Glasgow Centre for Virus Research, Institute of Infection, Inflammation and Immunity, College of Medical, Veterinary and Life Sciences, University of Glasgow, Glasgow, United Kingdom; 3 Animal Health Trust, Centre for Preventive Medicine, Lanwades Park, Newmarket, United Kingdom; 4 Wellcome Trust Sanger Institute, Wellcome Trust Genome Campus, Hinxton, Cambridge, United Kingdom; 5 The Genome Analysis Centre, Norwich Research Park, Norwich, United Kingdom; 6 Department of Ecology and Evolutionary Biology, Princeton University, Princeton, New Jersey, United States of America; 7 Center for Infectious Disease Dynamics, Department of Biology, The Pennsylvania State University, Pennsylvania, United States of America; 8 Fogarty International Center, National Institute of Health, Bethesda, Maryland, United States of America; University of Texas at Austin, United States of America

## Abstract

Influenza viruses are characterized by an ability to cross species boundaries and evade host immunity, sometimes with devastating consequences. The 2009 pandemic of H1N1 influenza A virus highlights the importance of pigs in influenza emergence, particularly as intermediate hosts by which avian viruses adapt to mammals before emerging in humans. Although segment reassortment has commonly been associated with influenza emergence, an expanded host-range is also likely to be associated with the accumulation of specific beneficial point mutations. To better understand the mechanisms that shape the genetic diversity of avian-like viruses in pigs, we studied the evolutionary dynamics of an Eurasian Avian-like swine influenza virus (EA-SIV) in naïve and vaccinated pigs linked by natural transmission. We analyzed multiple clones of the hemagglutinin 1 (HA1) gene derived from consecutive daily viral populations. Strikingly, we observed both transient and fixed changes in the consensus sequence along the transmission chain. Hence, the mutational spectrum of intra-host EA-SIV populations is highly dynamic and allele fixation can occur with extreme rapidity. In addition, mutations that could potentially alter host-range and antigenicity were transmitted between animals and mixed infections were commonplace, even in vaccinated pigs. Finally, we repeatedly detected distinct stop codons in virus samples from co-housed pigs, suggesting that they persisted within hosts and were transmitted among them. This implies that mutations that reduce viral fitness in one host, but which could lead to fitness benefits in a novel host, can circulate at low frequencies.

## Author Summary

The latest human influenza pandemic highlights the ability of influenza viruses to jump species barriers and emerge in new hosts, as well as the role of pigs in generating viruses with pandemic potential. The mutational power of influenza virus, caused by intrinsically error-prone viral polymerases, has been directly linked to viral emergence, as adaptive mutations present in the reservoir host are likely to be key to the evolution of sustained transmission in new hosts. Hence, studying how mutations are generated, maintained and transmitted in and among pigs is critical to understanding how novel viruses could emerge. Here we characterized the evolution and mutational spectra of influenza virus populations within naïve and vaccinated pigs linked by natural transmission, by analyzing multiple viral sequences obtained at different times post-infection. We show that the genetic make-up of influenza viruses in pigs is highly dynamic: the frequency of particular mutations, including those that could potentially alter host specificity or result in vaccine escape, fluctuated markedly, including one rapid fixation event. We also show that co-infections are common and multiple viruses – even defective ones – were transmitted between pigs despite being vaccinated. Our results provide empirical evidence of the complex dynamics of influenza viral populations in pigs and provide insight on the evolutionary basis of RNA viral emergence.

## Introduction

Influenza viruses are archetypical emerging viruses, as illustrated by the four human pandemics that have taken place since 1918. Although the natural reservoir of influenza viruses is wild waterfowl, the establishment of human lineages derived directly from birds is rare. The pig is therefore thought to play an important role in the adaptation of avian viruses to humans [1]. Despite the ongoing debate over whether the 1918 pandemic virus was transferred into humans directly from birds or if the pig was an intermediate host [2], the ecological importance of the latter in the generation of pandemic viruses is underscored by the latest H1N1 human pandemic [3], [4].

Although the 1957 and 1968 pandemics provide compelling evidence for the importance of segment reassortment in influenza emergence [5], [6], this process is not always a necessary requirement for the establishment of a novel lineage in a new host population. In particular, the emergence of Eurasian avian-like swine influenza virus (EA-SIV) in the late 1970's and the recent emergence of canine influenza virus (CIV) constitute examples of direct (i.e. without reassortment) host transfers from birds and horses into pigs and dogs, respectively [5], [7]. Clearly, during those host-switching events that do not involve reassortment, the rate at which adaptive mutations appear within individual animals is of critical importance.

Most of our knowledge on influenza virus evolution and emergence is based on the analysis of either partial or complete consensus sequence of genomes derived from samples collected in surveillance studies. Although fundamentally important, this only constitutes a partial picture of the processes that drive their epidemiology and evolution. Studies focusing on the drivers of viral diversity at other scales are therefore required to provide an integrated picture of influenza phylodynamics [8]. Recent studies have focused on the viral genetic diversity present within infected individuals using a variety of influenza viruses in diverse hosts [9]–[11]; this, in turn, provides an empirical framework for the quantitative analysis of host-pathogen interactions. Such studies are key to understanding how virus and host-associated traits influence the generation of viral genetic and phenotypic diversity and their impact on biological properties such as host range, antigenicity, antiviral resistance and virulence. Further, by studying intra-host viral diversity in the context of transmission experiments it is possible to infer the epidemiological consequences of within-host evolution by examining how transmission bottlenecks mediate the structure and extent of viral genetic diversity in the recipient host.

Studies that explore the within-host evolutionary dynamics of swine influenza viruses in pigs are lacking. EA-SIVs were first detected in 1979 [5], although it has been estimated that they may have originated as early as 1963 [12]. As noted above, this lineage is thought to have originated from a direct host-switch transfer from avian influenza A viruses. After its first isolation in 1979, EA-SIV became enzootic among pig populations in Western Europe and Asia and replaced the classical swine lineage that had been circulating for decades [13]. Of note, the neuraminidase (NA) and matrix (M) gene segments of the recently emerged human H1N1/2009 were derived from the EA-SIV lineage [3], [4].

The influenza hemagglutinin (HA) is a major surface glycoprotein that binds to host-cell receptors, and is also the main target for neutralizing antibodies [14]. As influenza infection results in partial cross-protection against novel variants, the HA is subject to strong immune selection. While mutations at antigenic sites can result in antigenic drift, amino acid changes at the receptor-binding domain (RBD) can result in expanded host-range [15], [16]. To determine the evolutionary dynamics of an Eurasian avian-like swine influenza virus in its natural host and how prior immunity impacts the mutational spectra of viral populations, we examined the intra- and inter-host genetic variation of the hemagglutinin 1 (HA1) gene of A/swine/England/453/2006 (H1N1) from a recent transmission study that included naïve and vaccinated pigs [17].

## Results

### Infected pigs exhibit high levels of within-host viral diversity

**Figure 1:**
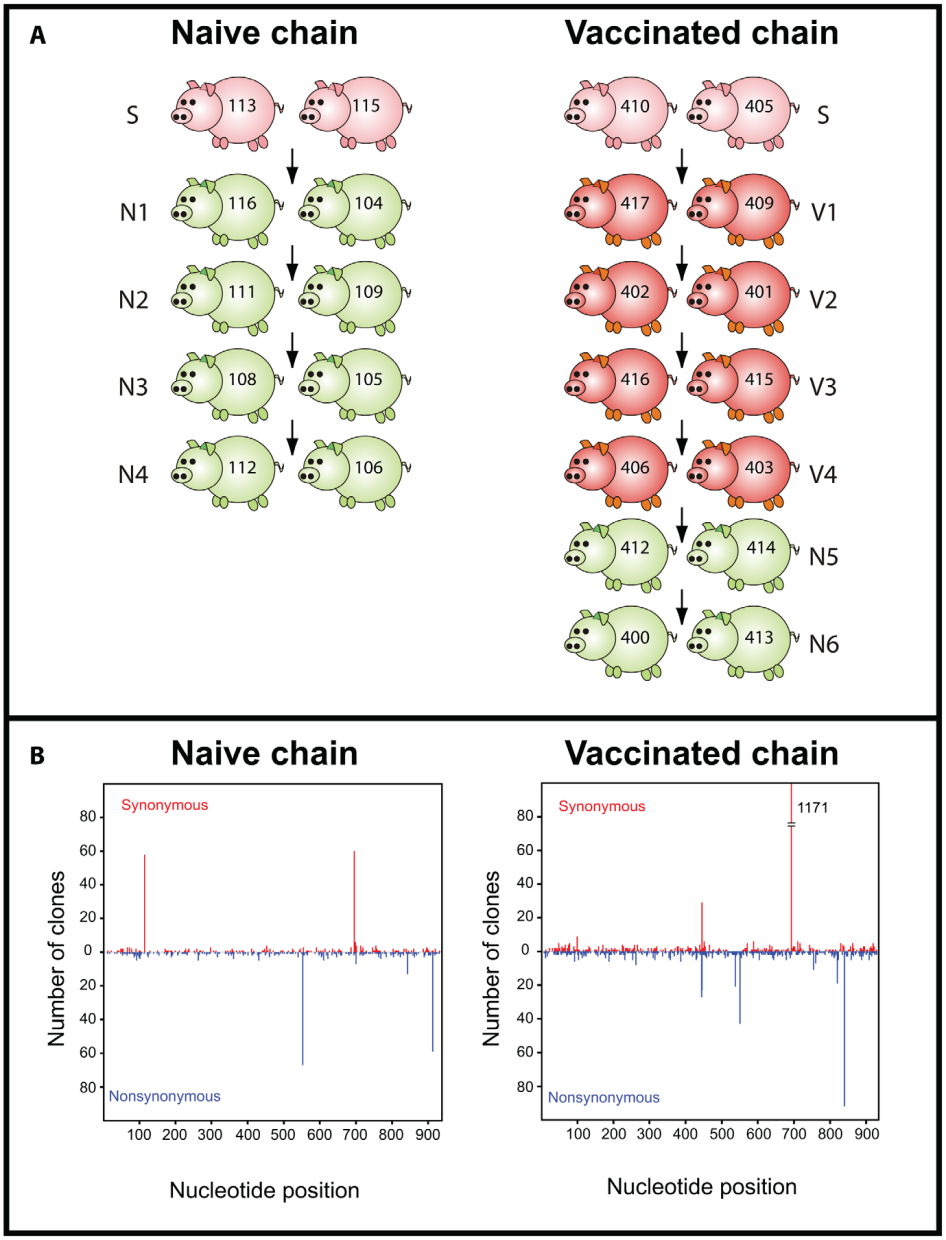
Layout of the transmission studies. Seeder pigs (S) were experimentally infected with A/swine/England/453/2006 and are shown in pink. Naïve pigs (N) are shown in green and vaccinated (V) pigs are shown in red. Arrows indicate the direction of transmission among pairs. Numbers on each pig refer to unique identifiers. (B) Nucleotide position and absolute frequency of synonymous and nonsynonymous mutations relative to the reference sequence. Each panel is shown below the corresponding study.

We examined the intra-host genetic variation of influenza virus present in daily nasal swabs obtained from two previously published transmission studies [17]. The “naïve” study consisted of a transmission chain among pairs of naïve pigs, while the “vaccinated” study involved the use of both naïve and vaccinated pigs (the latter immunized with an heterologous commercial bivalent vaccine containing A/New Jersey/8/76 [H1N1] and A/Port Chalmers/1/73 [H3N2]). This commercial vaccine is most broadly used in Europe. It was chosen to recreate the immune status of vaccinated pigs in the field as this would be the immune pressure that circulating viruses could face in nature. An outline of the experimental design is illustrated in Figure 1A. We generated 50 individual data sets, each derived from daily nasal swabs, and containing from 6 to 81 sequences of the first 939 nucleotides of the hemagglutinin 1 (HA1) gene. All antigenic sites and the receptor-binding domain were present in the HA1 region under study.

Within these data we observed 3129 mutations out of a total of 2,402,901 nucleotides sequenced, of which 684 were unique (i.e. occurred once in the whole data set). The estimated mutation frequency ranged from 2.8×10^−4^ (when only unique nucleotide changes were counted and assuming that repeated mutations resulted from viral replication) to 1.3×10^−3^ mutations per nucleotide site (when all mutations were considered independent events). The analysis of intra-host variation for the naïve and the vaccinated study is summarized in Tables 1 and 2, respectively. Synonymous (syn) and nonsynonymous (nonsyn) mutations were distributed throughout the HA1 segment without a clear regional clustering (Figure 1B). The overall frequency and distribution of mutations suggested that most of the observed nucleotide changes were due to random viral polymerase errors during virus replication. Consistent with this, the d_N_/d_S_ for the data set as a whole was 0.77 (95% CI = [0.72,0.84]), although we observed statistically significant evidence of positive selection (i.e. d_N_>d_S_) at codon positions 207 and 254. Interestingly, the former position lies within putative antigenic site Ca1 [18], [19].

**Table 1:** Analysis of intra-host variation of EA-SIV from the transmission experiment in naïve pigs.

Pig	Day^a^	No. of sequences	Total no. of mutations	No. of stop codons	Mean pairwise distance ± SE^c^	Mean d_N_/d_S_	No. of mutations in Ag sites
115*	2	57	56	0	0.0019±3.43E-05	1.08	7
	3	44	54	1	0.0024±4.95E-05	0.74	7
	4	48	41	0	0.0017±3.55E-05	0.84	4
113*	2	39	35	1	0.0018±4.06E-05	0.89	11
	3	33	18	0	0.0012±4.52E-05	3.38	5
	4	29	30	1	0.0022±9.15E-05	0.62	4
104	4	28	32	0	0.0023±7.03E-05	0.45	4
	5	77	36	2	0.0010±1.82E-05	0.87	6
	6	60	43	0	0.0015±2.90E-05	0.53	0
116	5	13	14	0	0.0013±0.00011	0.48	0
	6	6	12	1	0.0029±0.00016	0.64	0
109	7	55	69	0	0.0006±2.30E-05	1.75	57
	8	58	141	0	0.0009±2.36E-05	0.82	6
111	7	28	19	1	0.0014±7.06E-05	0.73	3
	8	40	26	1	0.0014±4.70E-05	1.17	3
105	9	34	12	2	0.0007±4.22E-05	1.35	5
108	9	38	27	0	0.0015±5.73E-05	0.71	1
	10	53	18	0	0.0007±2.70E-05	0.536	4
106	15	39	26	1	0.0014±3.98E-05	0.95	2
112	12	41	27	0	0.0014±3.90E-05	0.45	3
	15	48	22	1	0.0010±2.86E-05	0.68	1

*: Experimentally inoculated pig.

a Day after initiation of the transmission experiment.

c Standard error.

**Table 2:** Analysis of intra-host variation of EA-SIV from the transmission experiment in vaccinated pigs.

Pig	Day^a^	No. of sequences	Total no. of mutations	No. of stop codons	Mean pairwise distance	Mean d_N_/d_S_	No. of mutations in Ag sites
405*	2	81	65	0	0.0017±2.54E-05	1.49	13
	3	60	67	0	0.0023±3.77E-05	0.52	17
	4	65	86	0	0.0027±4.14E-05	1.14	24
	5	38	60	0	0.0026±5.28E-05	0.78	1
410*	3	68	71	0	0.0021±3.07E-05	0.67	10
	4	74	64	2	0.0018±2.91E-05	0.61	9
	5	60	76	1	0.0025±4.13E-05	0.65	7
409^b^	7	40	17	0	0.0009±3.93E-05	1.07	1
	9	49	13	0	0.0005±2.11E-05	0.56	2
417^b^	8	78	127	0	0.0013±2.19E-05	0.96	8
401^b^	9	53	76	0	0.0009±3.17E-05	0.69	3
	11	60	92	2	0.0012±2.54E-05	0.71	4
415^b^	13	60	128	0	0.0012±1.91E-05	0.59	13
416^b^	18	67	111	1	0.0014±2.94E-05	0.76	6
403^b^	18	49	67	0	0.0008±2.27E-05	0.89	0
406^b^	18	44	73	1	0.0014±3.98E-05	0.69	6
412^c^	19	15	21	1	0.0008±8.65E-05	0.16	2
	20	51	97	1	0.0030±5.73E-05	0.78	10
	21	74	130	1	0.0016±2.50E-05	1.34	8
414^c^	17	59	97	0	0.0014±2.70E-05	0.56	4
	19	71	117	0	0.0018±2.62E-05	0.72	6
	20	44	64	0	0.0010±3.78E-05	0.38	1
	21	38	51	0	0.0007±3.46E-05	1.24	2
	22	74	115	1	0.0012±2.28E-05	0.54	7
400^c^	19	65	100	0	0.0011±2.51E-05	1.60	3
	23	55	79	0	0.0009±2.64E-05	1.61	2
413^c^	22	64	93	0	0.0010±2.12E-05	0.65	3
	23	75	117	0	0.0012±2.18E-05	1.08	4
	24	60	97	0	0.0013±2.90E-05	0.68	6

*: Experimentally inoculated pig.

a Day after initiation of the transmission experiment.

b Vaccinated pig.

c Naïve pig.

### Within-host viral populations show a rapid turnover of mutations and fluctuating consensus sequences

**Figure 2:**
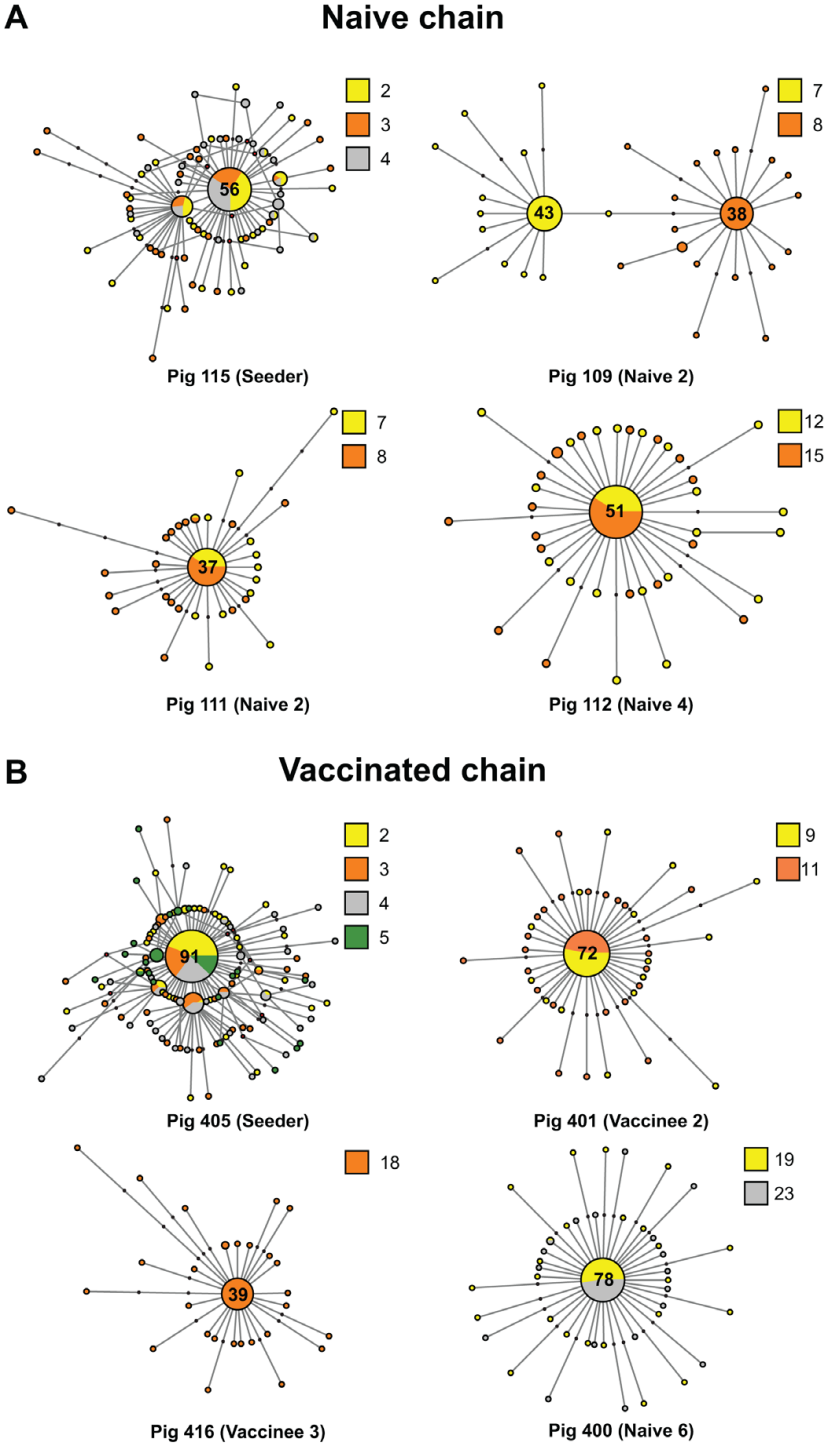
The mutational spectra of intra-host EA-SIV populations are highly dynamic. Median joining networks derived from representative individual pigs in the naïve (A) and vaccinated (B) studies. Each network was inferred by compiling sequences from multiple days. The number of sequences that constituted the consensus is indicated and circles are sized relative to their frequency in the data set. The identification number and the order of each pig the transmission chain is shown at the bottom of each panel. Colors indicate the day in which the sample was taken relative to the start of the study. Black dots along the branches indicate individual mutations relative to the sequence of the node from which they are derived. Cycles represent alternative potential evolutionary pathways.

**Figure 3:**
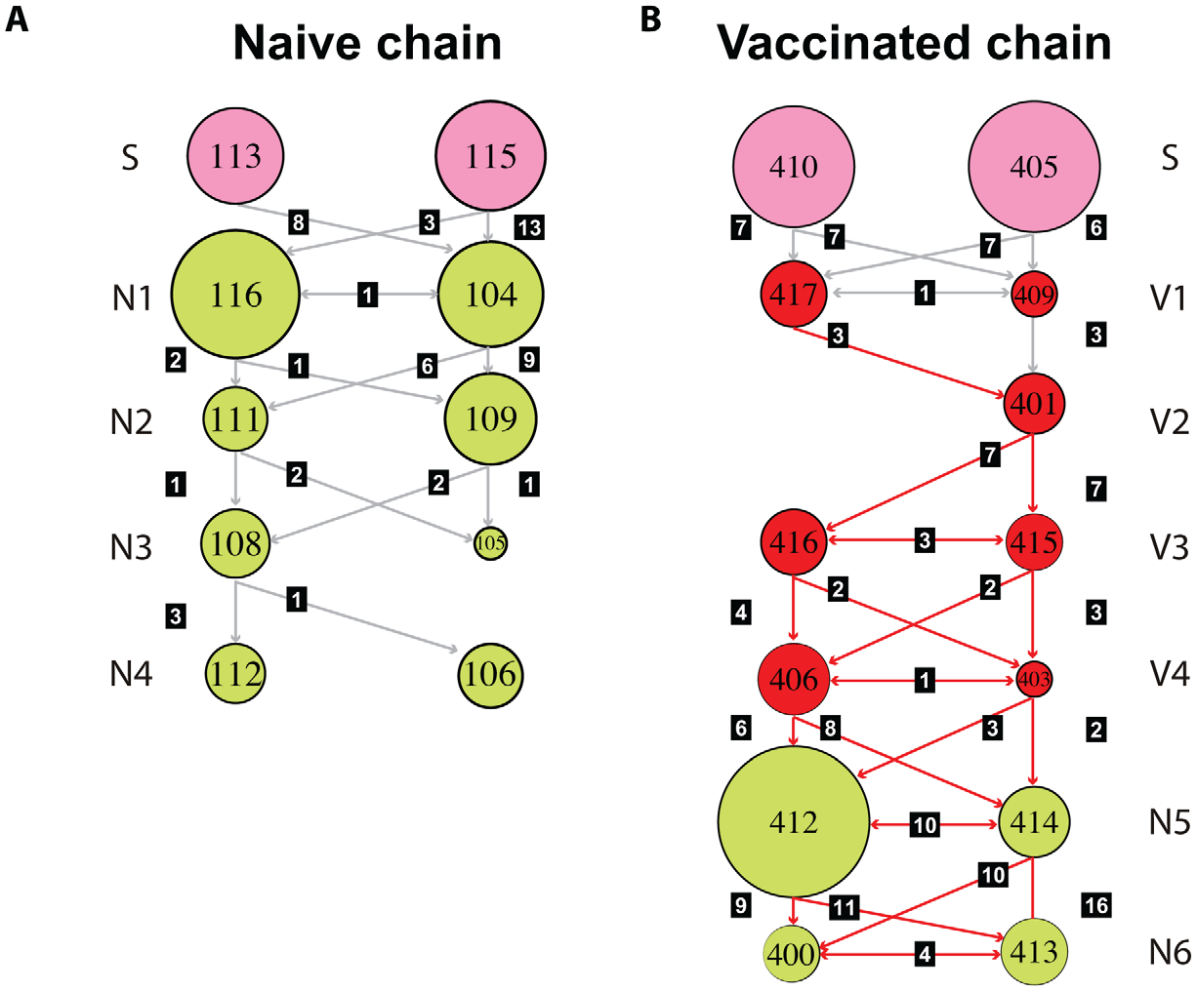
Transmission of multiple variants and mixed infections are common during infection of EA-SIV in pigs. Schematic representation of the number of shared mutations throughout the transmission studies in naïve (A) and vaccinated (B) pigs. Each circle represents a compiled data set for each pig (i.e. all the sequences derived from a pig along the course of infection), with the circle size being proportional to the mean pairwise distance of each data set. Seeder (S), naïve (N), and vaccinated (V) pigs are colored as shown in Figure 1. The infection route and relative position in the transmission chain is indicated on the left. The identification number of each pig is shown within each circle. The number of shared mutations between any two pigs is shown for in black boxes for each link in the chain. Grey arrows indicate that the consensus sequence of the viral population is the same as the reference whereas red arrows indicate the transmission of a fixed mutation (A696, see text). For full details of the shared mutations see Figure S3.

Daily viral populations were composed of a mixture of genomes closely related to a predominant or consensus sequence. However, the consensus was dynamic due to marked changes in the relative frequency of mutations within viral populations. For example, pig 109 in the naïve chain exhibited two different consensus populations on the two days that it was sampled (Figure 2a): on day 7 the consensus exhibited a nonsynonymous mutation at position 553 (Asn168Asp in the mature HA1), whilst on day 8 the majority of the sequences displayed two mutations: A696G (syn) and G914A (nonsyn, Ser288Asn), which in turn were different from the predominant sequence in all the previous animals. Interestingly, Asn168Asp is located at antigenic site Ca1, likely altering the overall antigenicity of that viral population on that day. This mutation is transmitted from pig 109 to pig 105 (Figure 3). Notably, however, these mutated consensus sequences were not fixed down the transmission chain. A median joining network for the naïve chain as a whole is shown in Figure S4.

Most striking of all, in the vaccinated chain synonymous mutation A696G (which was present at low frequency in three out of the four seeder pigs) became dominant in pig 417 and was then fixed along the transmission chain (Figure 3 and Figure S5). This is the only fixation event in our study. The reason why A696G displayed a transient high frequency in the naïve chains but was fixed only in the “vaccinated” transmission chain is unclear, but its appearance in both strongly suggests that this synonymous mutation has a marked impact on viral fitness.

### Mutations that could alter host range and antigenicity are likely to be transmitted

As it has been hypothesized that pigs act as “mixing vessels” in which avian viruses adapt to infect humans, we searched for mutations that could affect host-range. Accordingly, we detected two independent nonsynonymous mutations at amino acid position 133 within the receptor-binding domain (RBD). While Thr133Ile was observed in two consecutive days in naïve pig 104, Thr133Ala was observed in two pigs (410 and 401) in the vaccinated chain. Of note, when we examined this site at the epidemiological scale, the only residues observed were Thr and Ser, suggesting that Ile133 and Ala133 likely have a major impact on fitness, including altering host range. We also detected another amino acid change in the RBD: His180Arg was present in multiple animals along the transmission chain that included vaccinated pigs. An analysis of 2091 publicly available HA1 sequences from swine H1 viruses (Dataset S1) reveals that His180 is strictly conserved among them, and in 3671 HA sequences from a diversity of species only one isolate from mallards exhibited Asp180 and two isolates from humans displayed Pro180 (Dataset S2).

Similarly, we detected nonsynonymous changes at antigenic sites in both data sets (Tables 1 and 2). Interestingly, some of these were observed along multiple days and/or in different animals suggesting that they have been transmitted among them (Tables S1 and S2). For example, mutation A758G (Gln236Arg, Ca1 site) was present in pigs 401, 405, 412, 415 and 416 in the vaccinated study (Table S4), whereas mutation T623C (Leu191Pro, Sb site) was present in pigs 104, 108, 112 and 115 of the naïve study (Table S3). Moreover, we detected up to three mutations at antigenic sites in individual sequences. For example, a single sequence derived from naïve pig 111 displayed three mutations at antigenic sites (G270A, A607C, and A619G), two of which resulted in amino acid changes at antigenic sites Sb and Cb. Similarly, a sequence derived from vaccinated pig 417 exhibited two nonsynonymous mutations (A272G and G634A) located at antigenic sites Cb and Sb, respectively. These results therefore suggest that significant antigenic variation can be generated along the course of infection even in the presence of pre-existing immunity.

Glycosylation of the HA can impact both antigenicity and receptor binding [20]–[22]. There are five predicted N-glycosylation sites (N-X-S/T) in the HA1 segment under study, and we detected 40 mutations that disrupted glycosylation motifs, of which only two (Asn11Asp, Asn23Asp, Table S4) were possibly transmitted since they were present in animals linked by direct contact (pigs 400–414 and 412–413, respectively). We also detected six mutations that created glycosylation sites but they all constituted singletons.

### Intra-host variation in naturally infected pigs is different from that of inoculated pigs

We inferred maximum likelihood (ML) and median joining (MJ) trees from sequences derived from individual animals. Both the ML and MJ trees from the animals that were naturally infected down the transmission chain displayed a characteristic star-like structure. In contrast, more complex trees with multiple branching events were inferred for the viruses sampled from the inoculated animals that seeded the transmission chains, and which are indicative of more complex intra-host evolution (Figure 2 and Figure S2). Hence, it is possible that the type of the infection (natural vs. experimental) could have an impact on the nature of intra-host viral evolution. In this particular case such differences could be due to the large inoculation dose used to ensure infection and/or to the appearance of egg-adaptive mutations in the inocula (as the virus was egg-grown).

### Swine influenza virus displays wide transmission bottlenecks

A previous study suggested that transmission bottlenecks for equine influenza virus (EIV) in co-housed horses are not particularly tight, based on the observation of shared mutations among different horses in a transmission chain [9]. An analysis of the current pig data resulted in a similar observation; in particular, we observed multiple clones sharing identical mutations among different pigs (Tables S3 and S4). Indeed, we observed distinct mutations in multiple links of the transmission chain, including sequences sharing two mutations in different pigs, mostly in the transmission chain that included vaccinated pigs (Table 3). For example, mutation T867C was detected in vaccinated pigs 401, 415 and 403 (all linked by direct contact) and was linked to mutation A696G in pigs 415 and 403. We also observed sequences sharing three mutations between pigs: C447T, A824G and G844A were all present in the same clone, derived from pigs 410 and 412 in the vaccinated chain. Although it is possible that some of these mutations arose *de novo* in different pigs, this is not likely for sequences sharing multiple mutations.

**Table 3:** List of mutations present in the same clones that were transmitted among pigs in both studies.

Study	Linked mutations	Pigs	Direct contact?
Vaccinated pigs	447 **824 844**	412^c^, 405^b^, 410^b^	Yes
	**446** 447 **844**	412^c^, 405^b^, 410^b^	Yes
	**446 844**	405^b^, 410^b^	Yes
	**540** a **844**	412^c^, 405^b^, 410^b^	Yes
	**446** 447	412^c^, 405^b^, 410^b^	Yes
	447 573	405^b^, 410^b^	Yes
	**188 844**	405^b^, 410^b^	Yes
	447 **844 913**	405^b^, 410^b^	Yes
	573 **913**	412^c^, 410^b^	No
	687 **844**	405^b^, 410^b^	Yes
	**824 844**	412^c^, 405^b^, 410^b^	Yes
	**644 646** 696	413^c^, 416	No
	447 **844**	412^c^, 405^b^, 410^b^	Yes
	**263** a **844**	412^c^, 405^b^	No
Naïve pigs	453 **914**	109^c^, 115^b^	No

Nonsynonymous mutations are shown in bold.

a Antigenic site.

b Inoculated pig.

c Pig infected through natural transmission.

NB. Mutations linked to A696G have not been included.

By including pairs of pigs in each link of the chain we were also able to test if mixed infections were common; if this was the case, recipient pigs would harbor mutations present in both donor pigs. Notably, in both transmission studies we observed that mutations were likely to have been transmitted from both donors to single recipients, even in vaccinated pigs, thereby supporting the hypothesis of loose bottlenecks despite the presence of prior immunity (Figure 3). A complete description of the transmitted mutations (i.e. mutation type, motif, detection in multiple links in the transmission chain) is provided in Figure S3.

### Defective variants are transmitted in both naïve and vaccinated pigs

One of the most striking observations of our study was that the detection of 12 and 11 sequences carrying stop codons in the naïve and vaccinated transmission chains, respectively (Tables 1 and 2). In addition, three stop codon mutations – C361T, C487T and G420A – were at a frequency >1, while C361T and G420A were present in successive days of pigs 111 and 412, respectively (Table S5). Furthermore, C361T, C487T, and G420A were also present in multiple animals (some of them linked by direct transmission, Figure S3, and Table S5). For example, we observed mutation C361T (Gln104Stop) on day 5 of naïve pig 104, again on days 7 and 8 of naïve pig 111 and then again on day 15 of naïve pig 106. The fact that pigs 104 and 111 were co-housed and that this mutation was detected on two consecutive days in the latter, strongly suggests it was maintained throughout the course of infection and further transmitted (although the possibility of appearing *de novo* cannot be excluded entirely). Similarly, mutation C487T (Arg146Stop) was present on days 3 and 6 of co-housed pigs 115 and 116, respectively. Hence, these findings suggest that influenza viruses carrying low-fitness or deleterious mutations can persist and even be transmitted between pigs.

### The impact of prior immunity on within-host viral populations

During the transmission experiments, vaccinated pigs shed less virus than naïve ones [17]. To assess the impact of vaccination on intra-host viral populations we compared the sequences derived from only naturally infected naive and vaccinated pigs. The mean pairwise distance (MPD) was greater in the naïve group (MPD_naives_: 0.0018, MPD_vaccinees_: 0.0013) and this difference was statistically significant (t = 74.3461, p<2.2e-16) although small in absolute terms. Likewise, the proportion of singletons was higher in naïve pigs than in vaccinated pigs (0.30 and 0.17, respectively, W = 159, p-value = 0.004). Notably, with the exception of mutation A696G, we detected persistent mutations - i.e. those present in multiple days of a single animal - only in naïve pigs across both studies (Tables S1 and S2). However, we did not find significant differences in selective pressures between the two groups as the d_N_/d_S_ values for naive and vaccinated pigs were 0.76 and 0.70, respectively.

Finally, we hypothesized that viral populations from vaccinated pigs would exhibit a greater proportion of mutations at antigenic sites – a function of immune selection – and that transmission bottlenecks in this group of pigs would be tighter. Unexpectedly, we did not detect differences between the two groups. Similarly, we did not detect significant differences in the proportion of transmitted nonsynonymous mutations for each transmission chain. However, more mutations were transmitted between vaccinated (n = 64) than in naïve co-housed pigs (n = 52) even though the number of possible transmissions was lower in the former group (12 vs 16 respectively, see Figure 3).

## Discussion

Understanding the biological mechanisms that shape viral genetic diversity is essential to unravel fundamental aspects of influenza evolution, such as the generation of antigenic variation and the successful adaptation to new host species. Within a phylodynamic framework, experimental studies on intra- and inter-host influenza evolution are critical to link the dynamic processes that shape viral phylogenies from individual hosts to epidemiological-scale meta-populations.

Here, we determined the genetic variation of an Eurasian avian-like influenza virus along two transmission chains, one that included only naïve pigs and another that included both naïve and vaccinated pigs. The choice of the virus was based on the fact that this lineage established in the pig population following a complete genome interspecies transfer from birds [5], such that mutation accumulation rather than reassortment is likely to be central to host adaptation.

### Intra-host viral variation

We detected changes in the frequency of variants during the course of infection, revealing a complex pattern of within-host evolution. Our observation of transient changes in the consensus sequence of daily viral populations is of particular importance because it highlights the time-frame in which genetic (and potentially antigenic) novelty can be generated. This result is also consistent with that observed for canine influenza virus in dogs [10].

The estimated mutation frequency was approximately one order of magnitude higher than that previously reported for equine and avian influenza viruses in their natural hosts [9], [11]. This is noteworthy given the fact that in those studies the methodology was similar to that applied here. It is theoretically possible that the genetic structure of avian-like viral populations would change when infecting mammals, possibly as a result of virus adaptation to the host. Parallel studies comparing the evolutionary dynamics of long host-adapted swine viruses in pigs, human viruses in humans, avian viruses in pigs and in birds (i.e. ducks or chickens) could be used to address this hypothesis. It is also clear that some of the observed mutations could result from RT-PCR errors. Although the total proportion of artifact mutations cannot be estimated due to the impossibility of directly determining the number of mutations introduced during reverse transcription, we had previously estimated that the PCR enzymes used here under our laboratory conditions could introduce up to one mutation every 25,600 nucleotides [9]. If this was the case, less than 3% of the observed mutations would have been the result of PCR errors. The possibility that such mutations will alter the distributions of the sequences examined over time and/or along the transmission chains is highly unlikely, as we have previously shown using a Bayesian statistical framework to analyze within-host viral populations [23].

### Inter-host variation

In contrast with our previous study on the intra- and inter-host evolutionary dynamics of EIV [9], a change in the consensus sequence (A696G) became fixed in the transmission chain that included vaccinated pigs. Fixation on such a rapid scale can only realistically be explained by positive selection of the A696G mutation, or as a result of a hitchhiking effect (i.e. A696G is linked to a beneficial mutation somewhere else in the genome). The fact that G696 became fixed along the vaccinated but not the naïve chain despite being detected is suggestive of selection due to differences in immune pressure, even though it is a synonymous change. Indeed, at the epidemiological scale, G696 is present in 2.7% of the H1 sequences, confirming that viruses carrying this mutation have circulated. More generally, observing the process of allele fixation *in vivo* within days is of fundamental importance because it shows how rapidly natural selection can act on influenza viruses. Although the precise function of the A696G mutation is unknown, it is reasonable to think that a similarly rapid evolutionary process could apply to cases of vaccine escape, enhanced virulence or expanded host-range.

### Comparison of selective pressures across scales

The estimated d_N_/d_S_ for the intra-host data set was higher to that calculated for swine influenza H1N1 at the epidemiological scale (d_N_/d_S_: 0.34, 95% CI = [0.345,0.346]), in agreement with other studies on within-host viral evolution [9], [10] and reflecting the fact that intra-host genetic diversity frequently contains transient deleterious mutations that have yet to be removed by purifying selection. Despite this, we also observed that trees inferred from sequences derived from inoculated animals exhibited more complex topologies from those inferred from animals that were infected through natural transmission. This was likely due to the large inoculum doses used to ensure infection, and hence the transmission of more lineages, and the growth of virus in eggs. The latter process is likely to select for adaptive mutations, which is probable as the inoculum used was an avian-like swine virus. As such, our results suggest that caution should be taken when studying intra-host evolution in experimentally infected animals since the combination of large inocula and adaptive mutations generated during virus growth (either in cell culture or in eggs) could lead to artificially altered mutational spectra. For example, recent work has shown that the number of transmitted variants is correlated with the inoculum dose in a rhesus macaque infection model for HIV [24].

### Loose transmission bottlenecks are present despite vaccination

The observation of multiple genetic variants transmitted between pigs is consistent with what we observed in the case of EIV in horses [9]. However, the observation of loose transmission bottlenecks among vaccinated pigs is particularly striking as we had anticipated that the immune status of the host would impact on the size of the transmission bottleneck. Although uncertain, this could be a function of the phylogenetic (and likely antigenic) distance between the strains included in the vaccine and the challenge virus (Figure S1). Overall, our findings suggest that vaccination does not have a major effect on the genetic structure of intra-host viral populations through immune selection, nor on the size of transmission bottlenecks, at least for the combination of challenge virus and vaccine used in this study. Further experiments using homologous challenge and vaccine virus and different contact methods are required to address this point. Moreover, that we repeatedly detected the same mutations in recipient pigs as in both donor pigs indicates that mixed infections are common. The significance of this finding is enormous if we consider the structure of pig populations, where large numbers of piglets are often housed in warehouse-like buildings for several weeks until they reach their target weight at approximately 22–30 weeks. Although the all-in-all-out swine production system minimizes the transmission between groups of pigs, our results show that very high levels of genetic variation could be generated during growing/finishing stages even in vaccinated herds. Indeed, vaccination could result in undetected virus circulation as vaccinated pigs are likely to show very mild clinical signs of disease.

Also of note was the observation that mutations detected on multiple days were present in naïve but not in vaccinated pigs, consistent with similar studies we have performed in vaccinated horses (Murcia and others, unpublished). This could be due to a more efficient viral clearance in vaccinated pigs. Alternatively, since viral shedding was lower in this group [17], it is possible that persistent mutations were present but undetected. This would suggest that minor subpopulations could persist and be transmitted even in the presence of prior immunity, thus allowing natural selection to act more rapidly. Future work using ultra-deep sequencing technologies will address this issue.

One of our most notable observations of this study was that stop codon mutations, which are presumably defective, were both present within individual pigs and also transmitted among them. To the best of our knowledge, this is the first observation of the transmission of defective influenza viruses *in vivo*, although it has been reported in other RNA viruses [25]. In theory this could be achieved by trans-complementation, a mechanism that has been described during influenza replication *in vitro* [26], and implies that co-infection of single cells is a common feature during infection *in vivo*. This observation may have important implications for viral emergence, since it clearly means that low fitness mutations can be maintained within a host population, flattening the fitness valleys that separate donor and recipient hosts [27], such that mutations that are deleterious in the donor host may be advantageous in the recipient host.

In sum, the combination of loose bottlenecks, mixed infections, rapid allele fixation, common cellular co-infections and trans-complementation observed in this experimental study not only reveals the complex mechanisms at work during influenza evolution, but also provides a mechanistic framework to better understand the evolutionary basis of viral emergence.

## Materials and Methods

### Transmission experiments in pigs

The transmission studies from which the samples were obtained have been published previously [17]. All animal work was done under GB Home Office license following full ethical approval.

Naïve transmission study: five- to six-week-old piglets seronegative to influenza viruses of the H1N1, H1N2 and H3N2 subtypes were used. Two “seeders” were inoculated intranasally with 10^6.8^ EID_50_ of A/swine/England/453/2006. Upon confirmation of virus excretion using the Directigen test, two other piglets (N1) were introduced into the same pen. Upon detection of virus excretion in N1 pigs, the seeders were removed and two further piglets were introduced into the pen. This procedure was repeated in order to establish a transmission chain (Figure 1A). Nasal swabs were collected for up to four days after infection or contact, immersed in viral transport medium (VTM, PBS supplemented with 2% tryptose phosphate buffer broth, 2% penicillin/streptomycin and 2% amphotericin B), vortexed, aliquoted and stored at −80°C.

Transmission in vaccinated pigs: piglets were vaccinated with two doses of Gripovac, the first one at the age of four-to-five weeks and the second dose four weeks later. The viral strains contained in the vaccine (A/New Jersey/8/76 [H1N1] and A/Port Chalmers/1/73 [H3N2]) are different lineages from the challenge virus. Vaccinated pigs were tested by hemagglutination inhibition (HI) at regular intervals until the antibody titers reached a target value of ≤40 HIUs in order to allow natural infection. Vaccinated pigs reached the target antibody levels when they were approximately five months old. Two unvaccinated pigs were inoculated with 10^6.1^ EID_50_ of A/swine/England/453/2006 as described above. Nasal swabs were collected on a daily basis and an aliquot was RNA-extracted and subject to qPCR for virus detection on the same day. Upon detection of virus shedding the transmission chain was established as described above. Vaccinated pigs entered the transmission chain in order of antibody titer (lower first). Two pairs of naïve pigs were added to the end of the chain when virus shedding could no longer be detected in vaccinated pigs (Figure 1A). Details on the kinetics of viral shedding, clinical signs and gross lesions can be found in [17].

### Clonal sequencing of HA1 from nasal swabs

RNA was extracted from 280 µl-aliquots of nasal swabs using the QIAamp viral RNA mini kit (Qiagen). A two-step RT-PCR was performed to amplify a segment of HA1 starting from the 5′ terminal region to nucleotide position 1115. Reverse transcription was performed in 20 ul-reactions and PCR products used as a template 5 ul of cDNA. For most samples, cDNA from a single RT-PCR reaction was sufficient to generate enough clones for sequencing. cDNAs of the viral genomic HA gene was generated using Superscript III reverse transcriptase (Invitrogen) and primer Bm-HA1 [28]. Reverse transcription was performed at 55°C for 90 min, followed by incubation at 70°C for 10 min. PCR amplification was performed using Platinum Taq High Fidelity (Invitrogen) using primers Bm-HA1 [28] and HA1115 (5′RCTGTCCATCCCCCCTCAATYAANCCYGCAAT 3′). PCR amplification was performed for 35 cycles (94°C for 15 sec, 51°C for 30 sec and 68°C for 2 min) after 2 min of initial denaturation at 94°C and followed by a final extension at 68°C for 10 min.

Samples with low copy numbers were amplified using a hemi-nested PCR. The first PCR was performed using universal primers for the amplification of the full length HA gene [28], followed by a second reaction as described above. PCR products were gel-purified using the QIAquick Gel Extraction Kit (Qiagen) and further cloned using the Zero-Blunt TOPO PCR Cloning kit for sequencing (Invitrogen) following the manufacturer's instructions. Clones were sequenced at the Wellcome Trust Sanger Institute using fluorescent sequencing chemistry and ABI 3730xl capillary sequencers.

### Bioinformatic analysis

Forward and reverse sequencing reads from each clone were trimmed of vector sequence and poor quality regions. Reads with an average Phred quality score below 20 were rejected. We merged the forward and reverse reads into a single contig using the consensus HA1 sequence of stock A/swine/England/453/2006 (kindly provided by Ian Brown) as a framework reference. The alignment, assembly and analysis were performed using a bioinformatics tool specifically developed for mutation detection in viral sequences (Ramirez-Gonzalez, Hughes and Caccamo, in preparation). This software aligns the forward and reverse reads to the provided reference using a Smith-Waterman approach followed by a base-by-base inspection of the alignment. For every mismatch, it calls a mutation in the sample if the quality Phred score in the sequencing read is above 25 (this threshold is a parameter in the analysis). If the score is below the threshold it is assumed that the mismatch was induced by a sequencing error and the base in the provided reference is selected for the consensus. If the sequencing reads overlap, the base with the best quality is selected for the comparison with the reference. This analysis also computes the mutation type (i.e. whether they induce synonymous, nonsynonymous or missense alleles), which is reported by the tool together with the assembled contig. For the analysis reported here we only considered contigs longer than 935 nt and sequences with good quality insertions or deletions (introducing frameshifts) were also discarded.

### Evolutionary analysis

A total of 2559 intra-host EA-SIV sequences isolated from experimentally infected animals (GenBank accession numbers JQ520376–JQ522934), and 2091 epidemiological-scale publicly available H1 HA sequences from swine (Dataset S1, obtained from the Influenza Virus Resource [http://www.ncbi.nlm.nih.gov/genomes/FLU/FLU.html]) were collated. All sequences ran from the start codon of the HA open reading frame to nucleotide position 939. Sequence alignments were generated from the assembler output for the intra-host EA-SIV sequences, and using MAFFT [29] for the SIV epidemiological-scale sequences. Because of the very small genetic distances involved, the mean pairwise genetic diversity within each sample was estimated from the uncorrected pairwise distance matrix (*p*-distance) between taxa (available from the authors upon request). Maximum likelihood (ML) trees were estimated using PhyML [30] under the best-fit model of nucleotide substitution determined using MrAIC (http://www.abc.se/~nylander/mraic/mraic.html). For the very large data sets combining all sequences with the epidemiological-scale data, the phylogeny was estimated using RAxML [31] and the GTRGAMMA substitution model with 500 bootstrap replicates. Mean numbers of nonsynonymous (d_N_) and synonymous (d_S_) substitutions per site (ratio d_N_/d_S_) and the estimated 95% confidence intervals were estimated using the Single Likelihood Ancestor Counting (SLAC) algorithm available in the HyPhy software package [32]. Gaps were removed from the alignment prior to calculation of d_N_/d_S_. Finally, median joining networks were calculated from the sequence data using the median joining algorithm available in NETWORK 4.6.00 (http://www.fluxus-engineering.com/sharenet.htm).

## Supporting Information

Dataset S1 **2091 publicly available HA1 sequences from swine H1 viruses (fasta format).**
(TXT)

Dataset S2 **3671 publicly available H1 HA1 sequences derived from different species (fasta format).**
(TXT)

Figure S1 **Maximum likelihood phylogeny for HA1 clones from this study compared with the global phylogeny of H1N1 swine influenza viruses.** Sequences from this study are represented by a green triangle. The position of the vaccine strain in the tree is shown with an asterisk. Colored branches represent the distinct SIV phylogenetic groups. Bootstrap values are shown for key nodes and the horizontal branches are drawn to a scale of nucleotide substitutions per site.
(TIF)

Figure S2 **Median joining networks derived from individual pigs in the naïve and vaccinated studies.** Panel A: median joining networks from the naïve study. Panel B: median joining networks from the vaccinated study. Each network was inferred by compiling sequences from multiple days. The number of sequences that constituted the consensus is indicated and circles are sized relative to their frequency in the dataset. The identification number of each pig as well as the route of infection and the relative position in the transmission chain are shown on the top left of each panel and colors indicate the day in which the sample was taken relative to the start of the study. Black dots along the branches indicate individual mutations relative to the sequence of the node from which they are derived. S: seeder, N: Naïve, V: vaccinated. N/A: not applicable.
(PDF)

Figure S3 **Description of the shared mutations among different pigs.** Schematic representation of the shared mutations throughout the transmission studies in naïve (A) and vaccinated (B) pigs. Each circle represents a compiled data set for each pig (i.e. all the sequences derived from a pig along the course of infection), with the circle size being proportional to the mean pairwise distance of each data set. The identification number of each pig is shown within each circle. The shared mutations between any two pigs are shown for each link in the chain. The number of transmitted mutations is shown in black boxes. Linked mutations are shown in italics, mutations linked to A696G are shown with an asterisk. Mutations in black represent synonymous mutations, underlined mutations are those found in multiple links in the chain, non-synonymous mutations at a glycosylation site are shown in light blue and at antigenic sites are double underlined and shown in green, otherwise they are shown in red. The transmission of the reference sequence is not shown.
(TIF)

Figure S4 **Median joining network derived from the naïve transmission chain.** The network was inferred by compiling all the sequences from the pigs included in the naïve transmission chain. The number of sequences that reached high frequency is indicated and individual pigs are shown in different colors. Mutated nucleotides at specific positions are indicated for nodes that display a frequency >20. The position of nodes exhibiting sequences shared by different pigs was manually adjusted to improve clarity. Therefore, links between nodes are not drawn to scale.
(PDF)

Figure S5 **Median joining network derived from the vaccinated transmission chain.** The network was inferred by compiling all the sequences from the pigs included in the vaccinated transmission chain. The number of shared sequences that reached high frequency is indicated and individual pigs are shown in different colors. For the two main viral populations the nucleotide exhibited at position 696 is indicated. For clarity, the position of the nodes has been modified as in Figure S5.
(PDF)

Table S1 **Intra-host nonsynonymous mutations present in multiple days from the transmission experiment in naïve pigs.**
(DOCX)

Table S2 **Intra-host nonsynonymous mutations present in multiple days from the transmission experiment in vaccinated pigs.**
(DOCX)

Table S3 **Nonsynonymous mutations present in multiple pigs from the transmission experiment in naive pigs.**
(DOCX)

Table S4 **Nonsynonymous mutations present in multiple pigs from the transmission experiment in naive pigs.**
(DOCX)

Table S5 **Stop codons detected in multiple pigs.**
(DOCX)
