# Supplementary figures and images for: Evolution of an Eurasian Avian-like Influenza Virus in Naïve and Vaccinated Pigs
Source: PLoS Pathog. 2012 May 31;8(5):e1002730. doi: 10.1371/journal.ppat.1002730 (PMC3364949; doi:10.1371/journal.ppat.1002730)

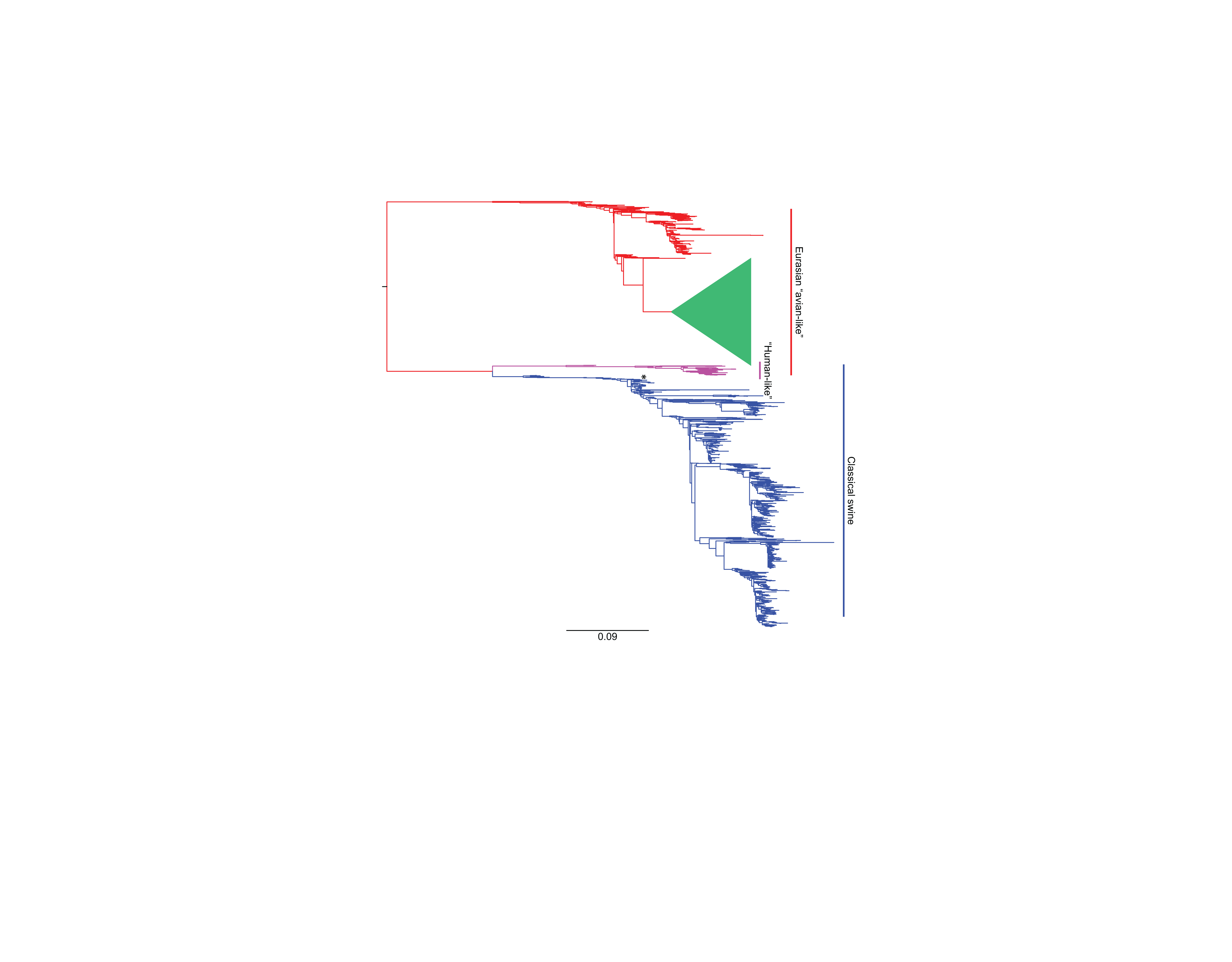

Supplement: Figure S1 — Maximum likelihood phylogeny for HA1 clones from this study compared with the global phylogeny of H1N1 swine influenza viruses. Sequences from this study are represented by a green triangle. The position of the vaccine strain in the tree is shown with an asterisk. Colored branches represent the distinct SIV phylogenetic groups. Bootstrap values are shown for key nodes and the horizontal branches are drawn to a scale of nucleotide substitutions per site. (TIF) [file ppat.1002730.s003.tif]

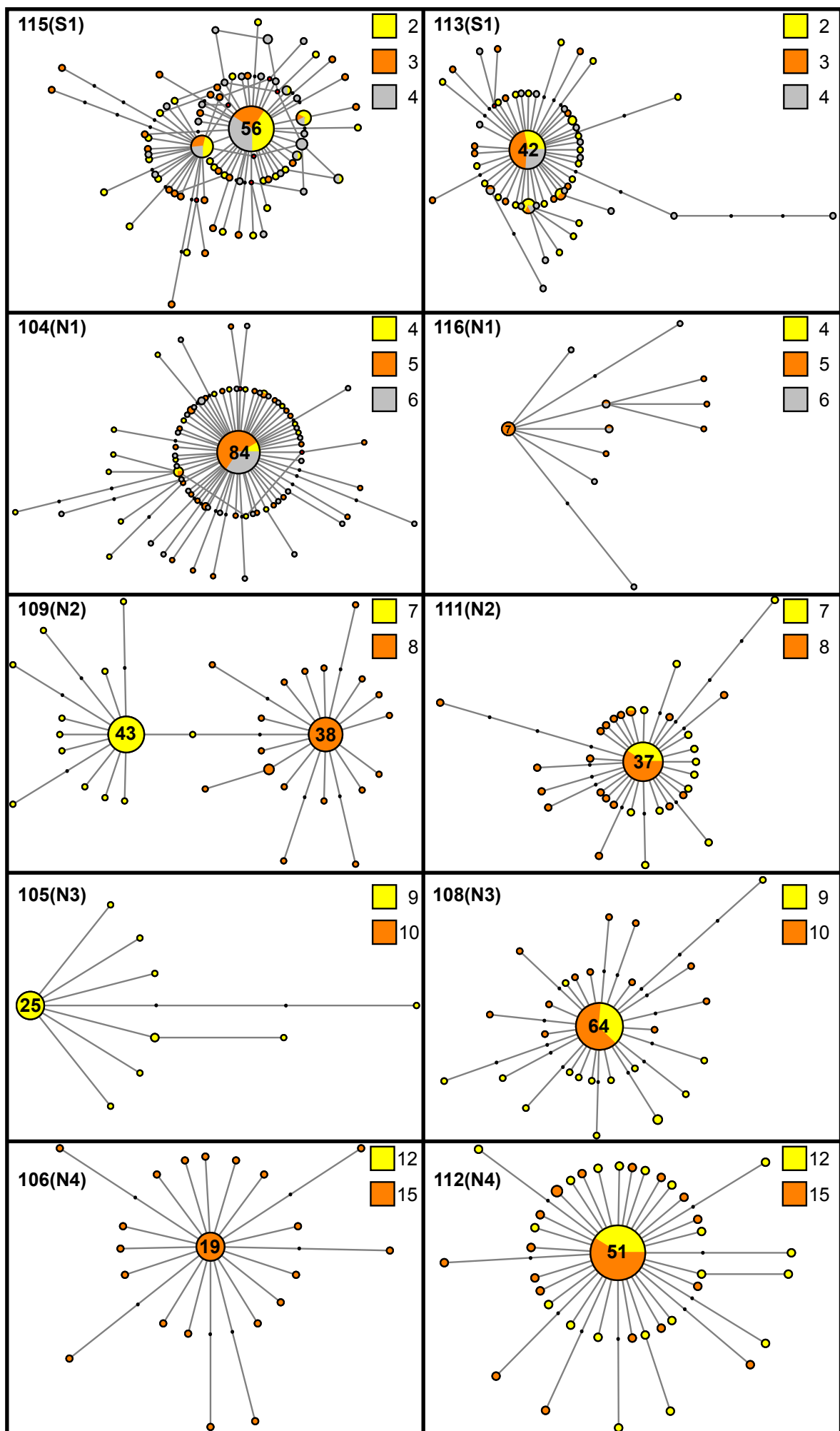

Figure 2(A)

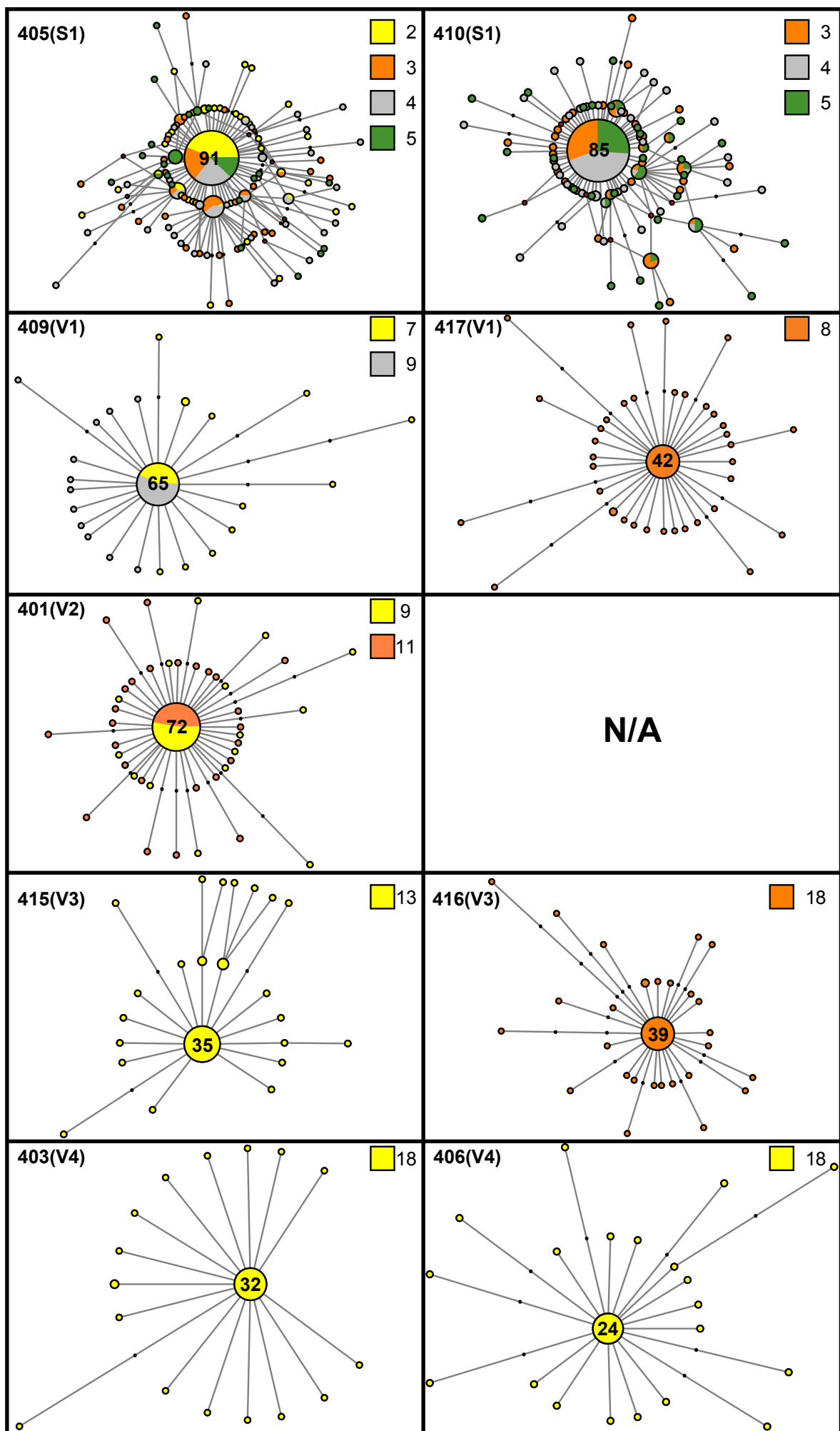

Figure 2(B)

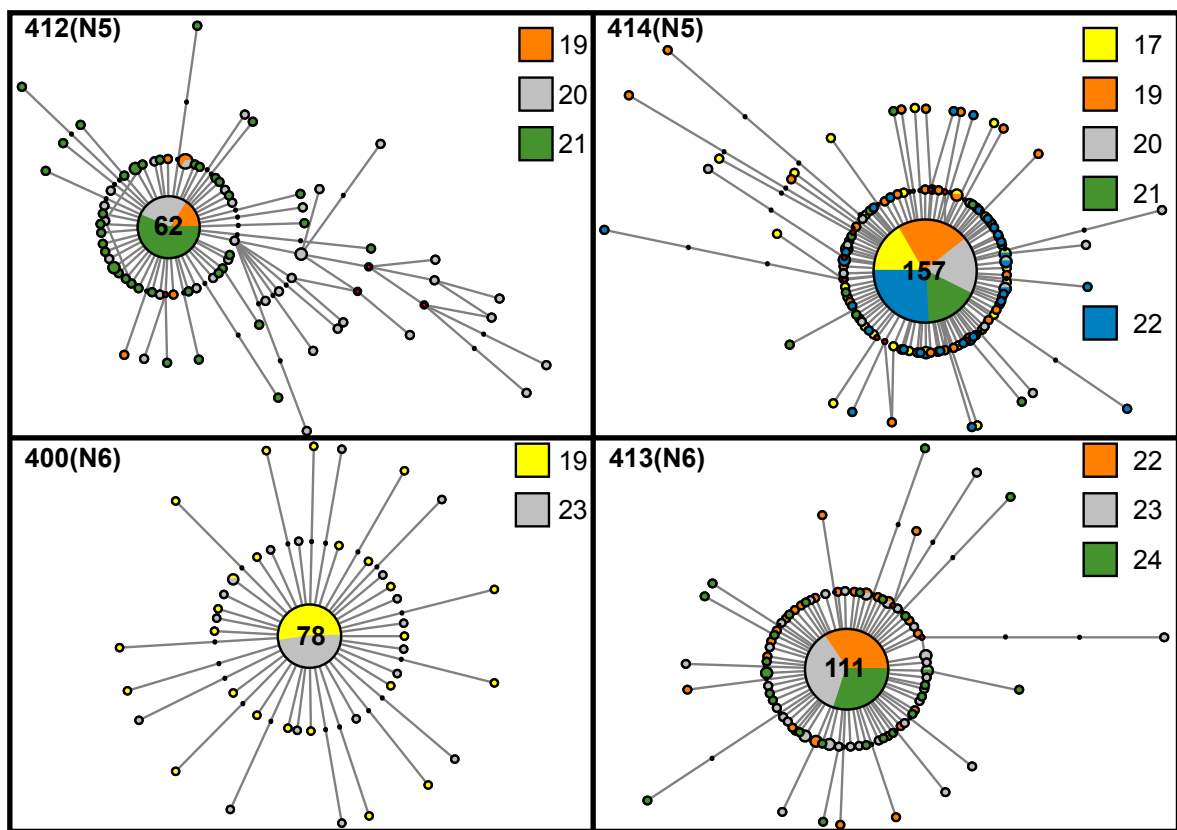

Figure 2(B) continued

Supplement: Figure S2 — Median joining networks derived from individual pigs in the naïve and vaccinated studies. Panel A: median joining networks from the naïve study. Panel B: median joining networks from the vaccinated study. Each network was inferred by compiling sequences from multiple days. The number of sequences that constituted the consensus is indicated and circles are sized relative to their frequency in the dataset. The identification number of each pig as well as the route of infection and the relative position in the transmission chain are shown on the top left of each panel and colors indicate the day in which the sample was taken relative to the start of the study. Black dots along the branches indicate individual mutations relative to the sequence of the node from which they are derived. S: seeder, N: Naïve, V: vaccinated. N/A: not applicable. (PDF) [file ppat.1002730.s004.pdf]

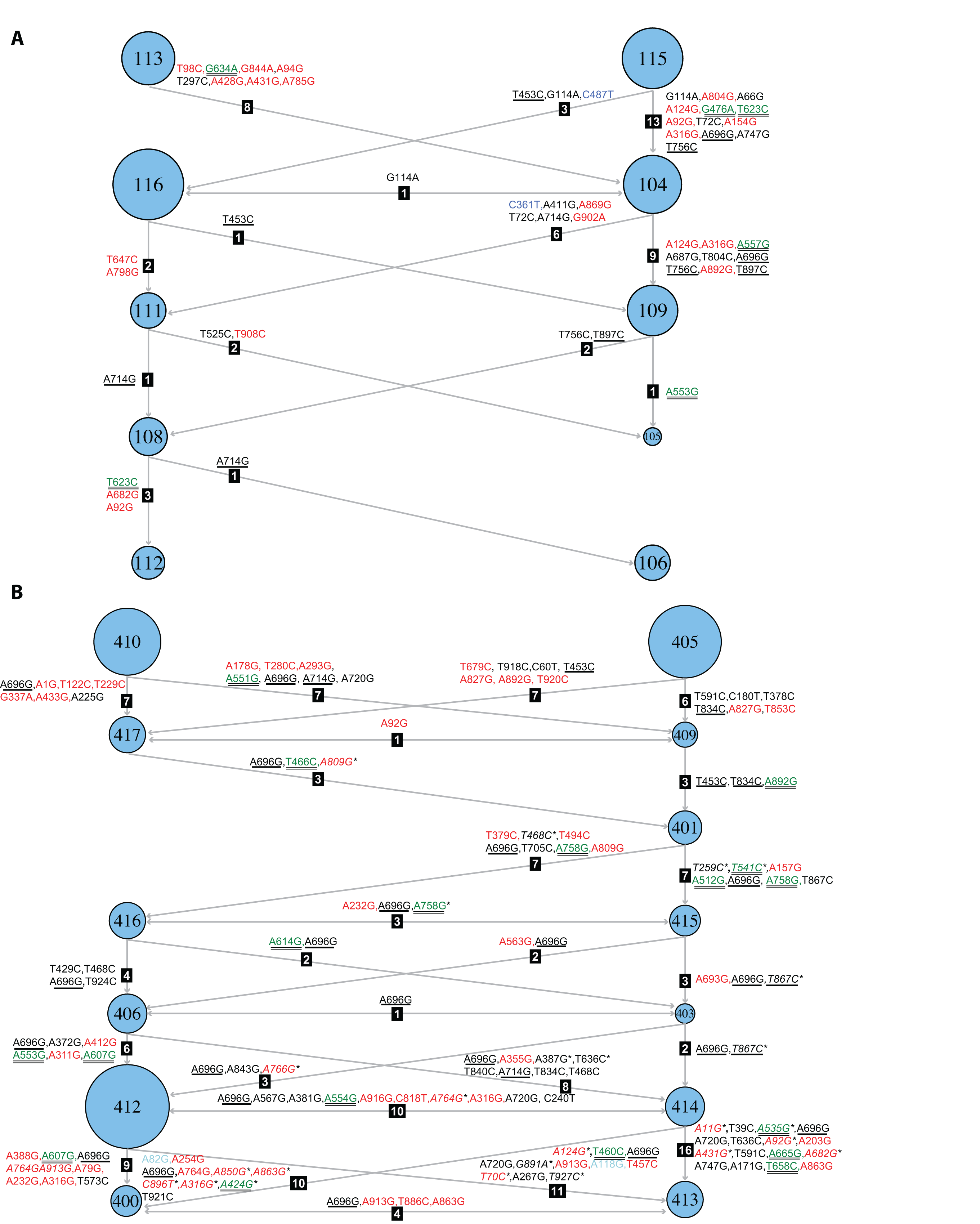

Supplement: Figure S3 — Description of the shared mutations among different pigs. Schematic representation of the shared mutations throughout the transmission studies in naïve (A) and vaccinated (B) pigs. Each circle represents a compiled data set for each pig (i.e. all the sequences derived from a pig along the course of infection), with the circle size being proportional to the mean pairwise distance of each data set. The identification number of each pig is shown within each circle. The shared mutations between any two pigs are shown for each link in the chain. The number of transmitted mutations is shown in black boxes. Linked mutations are shown in italics, mutations linked to A696G are shown with an asterisk. Mutations in black represent synonymous mutations, underlined mutations are those found in multiple links in the chain, non-synonymous mutations at a glycosylation site are shown in light blue and at antigenic sites are double underlined and shown in green, otherwise they are shown in red. The transmission of the reference sequence is not shown. (TIF) [file ppat.1002730.s005.tif]

Pig

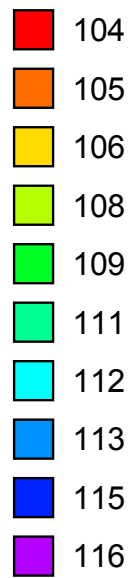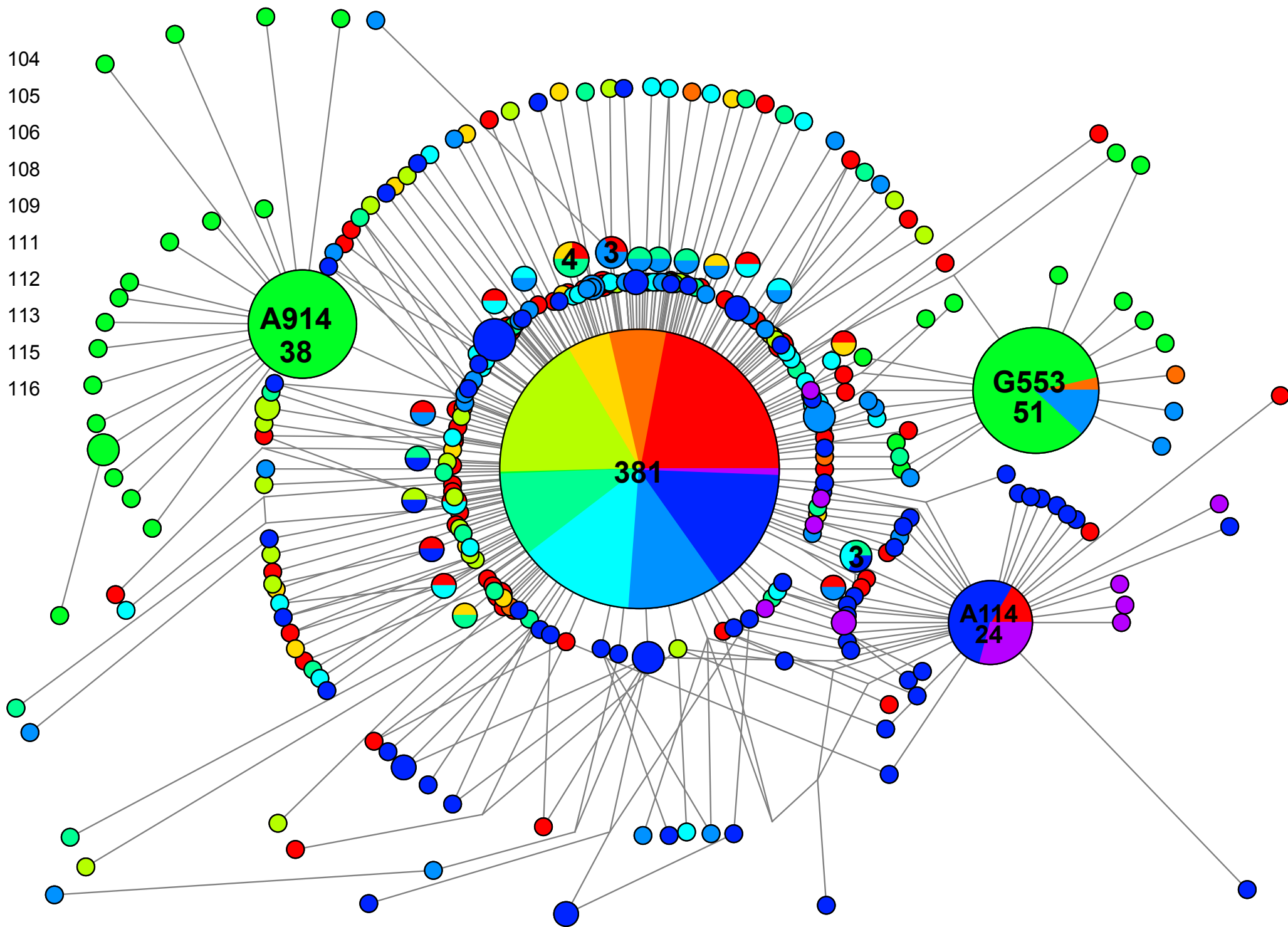

Supplement: Figure S4 — Median joining network derived from the naïve transmission chain. The network was inferred by compiling all the sequences from the pigs included in the naïve transmission chain. The number of sequences that reached high frequency is indicated and individual pigs are shown in different colors. Mutated nucleotides at specific positions are indicated for nodes that display a frequency >20. The position of nodes exhibiting sequences shared by different pigs was manually adjusted to improve clarity. Therefore, links between nodes are not drawn to scale. (PDF) [file ppat.1002730.s006.pdf]

Pig

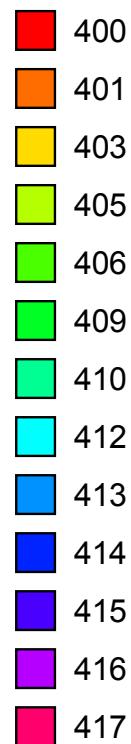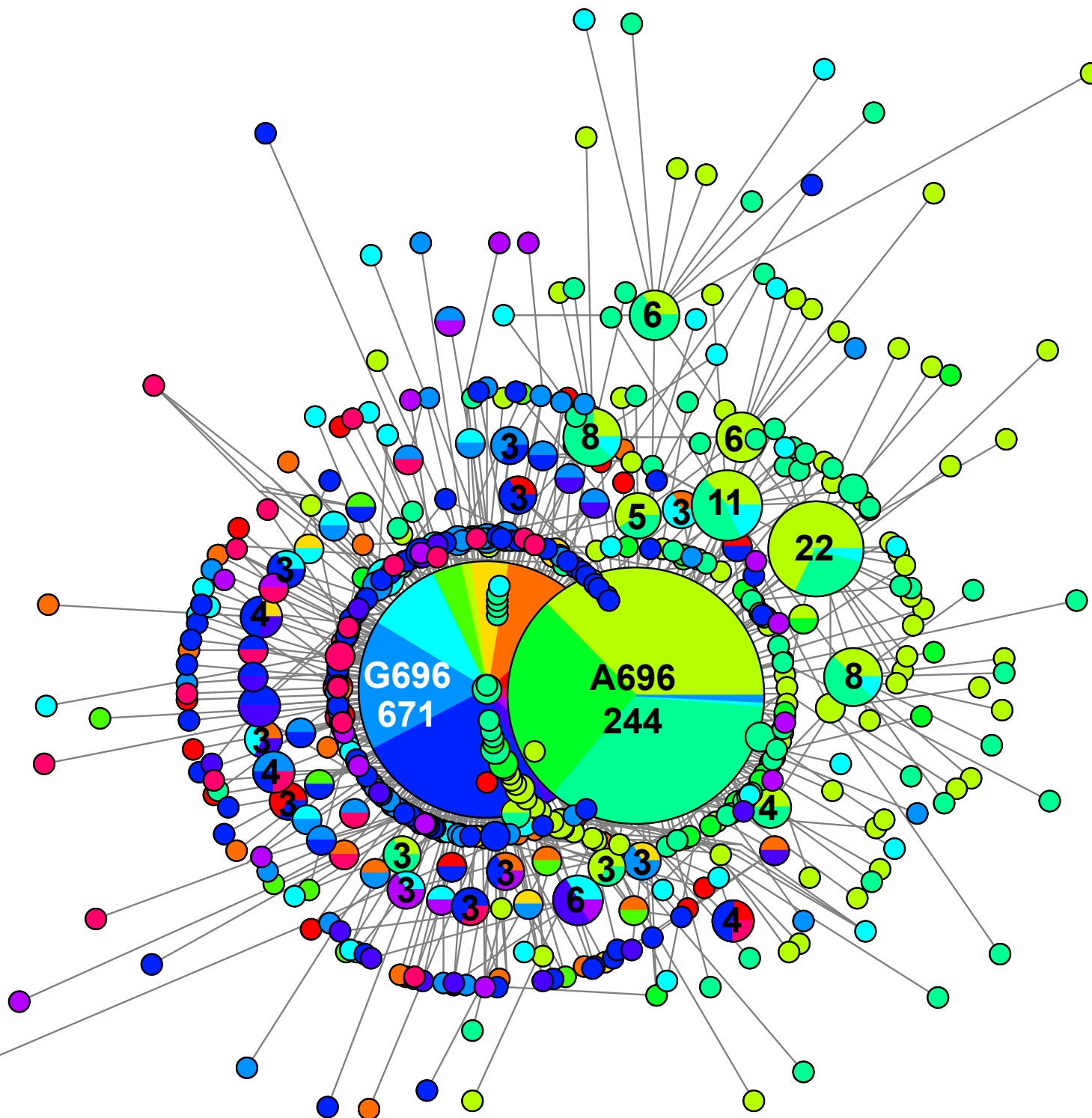

Supplement: Figure S5 — Median joining network derived from the vaccinated transmission chain. The network was inferred by compiling all the sequences from the pigs included in the vaccinated transmission chain. The number of shared sequences that reached high frequency is indicated and individual pigs are shown in different colors. For the two main viral populations the nucleotide exhibited at position 696 is indicated. For clarity, the position of the nodes has been modified as in Figure S5. (PDF) [file ppat.1002730.s007.pdf]
