# Supplementary material for: Evolution of an Eurasian Avian-like Influenza Virus in Naïve and Vaccinated Pigs
Source: PLoS Pathog. 2012 May 31;8(5):e1002730. doi: 10.1371/journal.ppat.1002730 (PMC3364949; doi:10.1371/journal.ppat.1002730)
Supplement: Table S1 — Intra-host nonsynonymous mutations present in multiple days from the transmission experiment in naïve pigs. (DOCX) [file ppat.1002730.s008.docx]

Table S1. Intra-host nonsynonymous mutations present in multiple days from the transmission experiment in naïve pigs

| Mutation^a^ | Motif | No. of pigs | Pig (Days) |
| --- | --- | --- | --- |
| A92G Asp14Gly | NA | 1 | 115^b^ (3,4) |
| A101G Asp17Gly | NA | 1 | 115^b^ (2,3) |
| A103G Thr18Ala | NA | 1 | 115^b^ (2,3) |
| T122C Val24Ala | NA | 1 | 115^b^ (2,4) |
| A232G Ile61Val | NA | 1 | 115^b^ (2,4) |
| C361T Gln104Stop | NA | 1 | 111^c^ (7,8) |
| A388G Arg113Gly | NA | 1 | 115^b^ (2,4) |
| A431G Glu127Gly | NA | 1 | 113^b^ (3,4) |
| C449T Thr133Ile | RBD | 1 | 104^c^ (4,5) |
| G540T Lys163Asn | AgSa | 1 | 115^b^ (2,4) |
| A553G Asn168Asp | AgCa1 | 1 | 113^b^ (2,3,4) |
| A605G Asp185Gly | AgSb | 1 | 115^b^ (2,4) |
| A611G Asp187Gly | AgSb | 1 | 113^b^ (2,3,4) |
| T647C Val199Ala | NA | 1 | 116^c^ (5,6) |
| G701A Arg217Lys | NA | 1 | 115^b^ (2,3,4) |
| A764G Asp238Gly | NA | 1 | 104^c^ (4,5) |
| G844A Val265Ile | NA | 1 | 115^b^ (2,4) |
| A890G Gln280Arg | NA | 1 | 113^b^ (3,4) |

^a^ Amino acid numbering based on mature HA1.

^b^ Inoculated pigs.

^c^ Pig infected through natural transmission.

RBD: Receptor binding domain. AgSa: Antigenic site Sa. AgCa1: Antigenic site Ca1. AgSb: Antigenic site Sb. NA: Not applicable.
