# Supplementary material for: Evolution of an Eurasian Avian-like Influenza Virus in Naïve and Vaccinated Pigs
Source: PLoS Pathog. 2012 May 31;8(5):e1002730. doi: 10.1371/journal.ppat.1002730 (PMC3364949; doi:10.1371/journal.ppat.1002730)
Supplement: Table S2 — Intra-host nonsynonymous mutations present in multiple days from the transmission experiment in vaccinated pigs. (DOCX) [file ppat.1002730.s009.docx]

Table S2. Intra-host nonsynonymous mutations present in multiple days from the transmission experiment in vaccinated pigs

| Mutation^a^ | Motif | No. of pigs | Pig (Days) |
| --- | --- | --- | --- |
| G188A Gly46Glu | NA | 2 | 405^b^ (4,5) 410^b^ (3,4,5) |
| T233C Ile61Thr | NA | 1 | 413^c^ (23,24) |
| A254G Asp68Gly | NA | 1 | 414 (19,20) |
| T263C Leu71Pro | AgCb | 2 | 412^c^ (20,21) 405^b^ (2,5) |
| A316G Thr89Ala | NA | 1 | 414^c^ (20,22) |
| A359G Glu103Gly | NA | 1 | 405^b^ (2,3,4) |
| A388G Arg113Gly | NA | 1 | 405^b^ (2,5) |
| G420A Trp123Stop | NA | 1 | 412^c^ (20,21) |
| A431G Glu127Gly | NA | 1 | 413^c^ (23,24) |
| C446A Ser132Tyr | NA | 2 | 405^b^ (2,3,4,5) 410^b^ (3,5) |
| C446T Ser132Phe | NA | 2 | 405^b^ (2,3,4,5) 410^b^ (3,4) |
| C447T Ser132Tyr | NA | 1 | 405^b^ (2,3) |
| T460C Ser137Pro | AgCa2 | 1 | 413^c^ (22,23) |
| A512G Lys154Arg | AgSa | 1 | 414^c^ (17,22) |
| G540T Lys163Asn | AgSa | 2 | 405^b^ (2,4) 410^b^ (3,4,5) |
| A553G Asn168Asp | AgCa1 | 2 | 405^b^ (2,3,4) 410^b^ (3,4,5) |
| A557G Lys169Arg | AgCa1 | 1 | 412^c^ (20,21) |
| T643C Tyr198His | NA | 1 | 405^b^ (2,4) |
| A644G Tyr198Cys | NA | 1 | 413^c^ (22,23) |
| A682G Thr211Ala | NA | 1 | 405^b^ (3,4) |
| A764G Asp238Gly | NA | 1 | 414^c^ (17,20,22) |
| T770C Ile240Thr | NA | 1 | 405^b^ (2,3) |
| A824G Asn258Ser | NA | 2 | 405^b^ (2,3,4,5) 410^b^ (3,4,5) |
| G844A Val265Ile | NA | 2 | 405^b^ (2,3,4,5) 410^b^ (3,4,5) |
| A850G Met267Val | NA | 1 | 400^c^ (19,23) |
| A863G Gln271Arg | NA | 1 | 414^c^ (17,19) |
| A890G Gln280Arg | NA | 1 | 405^b^ (2,3) |

^a^ Amino acid numbering based on mature HA1.

^b^ Inoculated pigs.

^c^ Pig infected through natural transmission.

AgSa: Antigenic site Sa. AgSb: Antigenic site Sb.AgCa1: Antigenic site Ca1. AgCa2: Antigenic site Ca2. AgCab: Antigenic site Cab.
