# Supplementary material for: Evolution of an Eurasian Avian-like Influenza Virus in Naïve and Vaccinated Pigs
Source: PLoS Pathog. 2012 May 31;8(5):e1002730. doi: 10.1371/journal.ppat.1002730 (PMC3364949; doi:10.1371/journal.ppat.1002730)
Supplement: Table S3 — Nonsynonymous mutations present in multiple pigs from the transmission experiment in naive pigs. (DOCX) [file ppat.1002730.s010.docx]

Table S3. Nonsynonymous mutations present in multiple pigs from the transmission experiment in naive pigs

| Mutation | No. of Pigs | | Pig (Days^a^) | |
| --- | --- | --- | --- | --- |
| A53G Asp1Gly | 2 | | 112(15) 113(2) | |
| T65C Val5Ala | 2 | | 113(3) 115(2) | |
| T85C Ser12Pro | 2 | | 112(12) 115(3) | |
| A88G Thr13Ala | 2 | | 109(8) 115(2) | |
| A92G Asp14Gly | 4 | | 104(6) 108(9) 112(15) 115(3,4) | |
| A94G Thr15Ala | 2 | | 104(6) 113(3) | |
| T98C Val16Ala | 3 | | 104(4) 112(12) 113(2) | |
| A101G Asp17Gly | 3 | | 111(7) 112(15) 115(2,3) | |
| A124G Thr25Ala | 3 | | 104(5) 109(7) 115(2) | |
| A154G Ser35Gly | 2 | | 104(6) 115(2) | |
| T175C Cys42Arg | 2 | | 108(9) 113(3) | |
| T191C Val47Ala | 2 | | 109(8) 113(3) | |
| T263C Leu71Pro* | 2 | | 109(8) 115(4) | |
| A265G Thr72Ala* | 2 | | 113(2) 115(2) | |
| A316G Thr89Ala | 3 | | 104(6) 109(7) 115(2) | |
| A347G Glu99Gly | 2 | | 104(4) 108(9) | |
| A355G Arg102Gly | 2 | | 106(15) 115(4) | |
| C361T Gln104Stop | 3 | | 104(5) 106(15) 111(7,8) | |
| A412G Thr121Ala | 2 | | 111(8) 115(3) | |
| C413T Thr121Ile | 2 | | 106(15) 113(2) | |
| A428G His126Arg | 2 | | 104(6) 113(2) | |
| A431G Glu127Gly | 3 | | 104(6) 108(10) 113(3,4) | |
| C461T Ser137Phe* | 2 | | 113(3) 115(3) | |
| G476A Arg142His* | 2 | | 104(5) 115(3) | |
| C487T Arg146Stop | 2 | | 115(3) 116(6) | |
| A553G Asn168Asp* | 3 | | 105(9) 109(7) 113(2,3,4) | |
| A557G Lys169Arg* | 2 | | 104(5) 109(7) | |
| A605G Asp185Gly* | 2 | | 113(2) 115(2,4) | |
| A611G Asp187Gly* | 2 | | 109(7) 113(2,3,4) | |
| A619G Ser190Gly* | 2 | | 111(7) 112(12) | |
| T623C Leu191Pro* | 4 | | 104(5) 108(9) 112(12) 115(4) | |
| G634A Ala195Thr* | 2 | | 104(4) 113(3) | |
| T647C Val199Ala | 2 | | 111(8) 116(5,6) | |
| A682G Thr211Ala | 2 | | 108(10) 112(12) | |
| C695T Ala215Val | 2 | | 104(4) 106(15) | |
| C722T Ala224Val | 2 | | 106(15) 109(8) | |
| A764G Asp238Gly | 2 | | 104(4,5) 112(15) | |
| T770C Ile240Thr | 3 | | 106(15) 113(2) 115(2) | |
| A785G Asn245Ser | 3 | | 104(6) 113(2) 115(2) | |
| A798G Ile249Met | 2 | 111(8) 116(6) | |  |
| T805C Trp252Arg | 3 | | 104(4) 109(7) 112(15) | |
| A824G Asn258Ser | 2 | | 113(4) 115(3) | |
| C833T Pro261Leu | 2 | | 109(7) 115(2) | |
| G844A Val265Ile | 3 | | 104(5) 113(4) 115(2,4) | |
| G859A Ala270Thr | 2 | | 108(9) 115(3) | |
| A863G Gln271Arg | 2 | | 104(6) 112(15) | |
| A869G His273Arg | 2 | | 104(6) 111(7) | |
| A890G Gln280Arg | 2 | | 113(3,4) 115(3) | |
| A892G Thr281Ala | 2 | | 104(5) 109(8) | |
| G902A Gly284Glu | 2 | | 104(5) 111(8) | |
| G904A Ala285Thr | 2 | | 111(8) 113(3) | |
| T908C Leu286Ser | 2 | | 105(9) 111(8) | |
| G914A Ser288Asn | 2 | | 109(8) 115(3) | |
| T920C Leu290Pro | 2 | | 111(7) 113(3) | |

* Antigenic site

^a^ Day after initiation of the transmission experiment.
