# Supplementary material for: Evolution of an Eurasian Avian-like Influenza Virus in Naïve and Vaccinated Pigs
Source: PLoS Pathog. 2012 May 31;8(5):e1002730. doi: 10.1371/journal.ppat.1002730 (PMC3364949; doi:10.1371/journal.ppat.1002730)
Supplement: Table S4 — Nonsynonymous mutations present in multiple pigs from the transmission experiment in naive pigs. (DOCX) [file ppat.1002730.s011.docx]

Table S4. Nonsynonymous mutations present in multiple pigs from the transmission experiment in vaccinated pigs

| Mutation | No. of pigs | Pigs (Day^c^) |
| --- | --- | --- |
| A1G Met1Val^§^ | 3 | 410(5) 413(24) 417(8) |
| A11G Lys4Arg^§^ | 4 | 401(9) 405(2) 413(22) 414(19) |
| T16C Phe6Leu^§^ | 2 | 416(18) 417(8) |
| G40A Ala!4Thr§ | 2 | 405(5) 414(19) |
| T44C Leu15Ser^§^ | 2 | 405(4) 414(19) |
| A46G Lys16Glu^§^ | 2 | 410(4) 414(17) |
| A47G Lys16Arg^§^ | 2 | 405(2) 414(20) |
| A55G Thr2Ala | 2 | 414(19) 417(8) |
| T70C Tyr7His | 3 | 405(5) 412(20) 413(24) |
| C73T His8Tyr | 2 | 401(11) 415(13) |
| A79G Asn10Asp | 2 | 400(23) 412(21) |
| A82G Asn11Asp^a^ | 2 | 400(19) 414(22) |
| A92G Asp14Gly | 4 | 409(7) 413(23) 414(22) 417(8) |
| A101G Asp17Gly | 2 | 410(4) 414(22) |
| A103G Thr18Ala | 3 | 401(11) 405(3) 414(19,22) |
| T110C Leu20Pro | 2 | 400(19) 401(11) |
| G112A Glu21Lys | 2 | 412(21) 416(18) |
| A113G Glu21Gly | 3 | 401(9) 410(5) 412(21) |
| A118G Asn23Asp^a^ | 2 | 412(21) 413(23) |
| T122C Val24Ala | 2 | 410(4) 417(8) |
| A124G Thr25Ala | 3 | 410(5) 412(21) 413(22) |
| A157G Ser36Gly | 3 | 400(19) 401(9) 415(13) |
| A164G Asn38Ser | 2 | 405(5) 413(22) |
| T175C Cys42Arg | 2 | 414(17) 416(18) |
| A178G Ser43Gly | 3 | 400(19) 405(5) 417(8) |
| G188A Gly46Glu | 2 | 405(4,5) 410(3,4,5) |
| T191C Val47Ala | 2 | 406(18) 409(7) |
| C194T Ala48Val | 2 | 400(19) 410(5) |
| A203G Gln51Arg | 2 | 413(23) 414(19) |
| A218G Asp56Gly | 2 | 414(17) 416(18) |
| A222G Ile57Met | 2 | 405(3) 413(24) |
| T229C Trp60Arg | 2 | 410(3) 417(8) |
| A232G Ile61Val | 5 | 400(23) 405(3) 412(21) 415(13) 416(18) |
| A254G Asp68Gly | 4 | 400(19) 410(3) 414(19,22) 415(13) |
| T263C Leu71Pro* | 3 | 405(2,5) 412(20,21) 416(18) |
| T280C Ser77Pro | 2 | 405(2) 417(8) |
| A293G Glu81Gly | 3 | 405(5) 413(23) 417(8) |
| A295G Thr82Ala | 2 | 400(23) 417(8) |
| A311G Asn87Ser | 3 | 406(18) 409(7) 412(21) |
| A316G Thr89Ala | 4 | 400(23) 412(21) 414(20,22) 417(8) |
| T322C Tyr91His | 2 | 403(18) 413(23) |
| G337A Ala96Thr | 2 | 410(4) 417(8) |
| A355G Arg102Gly | 4 | 401(11) 405(4) 406(18) 414(19) |
| A359G Glu103Gly | 2 | 405(2,3,4) 414(20) |
| T370C Ser107Pro | 2 | 413(24) 415(13) |
| T379C Ser110Pro | 2 | 401(11) 416(18) |
| A388G Arg113Gly | 4 | 400(19) 405(2,5) 410(5) 412(20) |
| A395G Glu115Gly | 2 | 414(19) 417(8) |
| A412G Thr121Ala | 2 | 406(18) 412(20) |
| G420A Trp123Stop | 2 | 401(11) 412(20,21) |
| A424G Asn125Asp* | 2 | 400(23) 414(19) |
| A431G Glu127Gly | 2 | 413(23,24) 414(21) |
| A433G Thr128Ala | 3 | 400(19) 410(5) 417(8) |
| C446A Ser132Tyr | 3 | 405(2,3,4,5) 410(3,5) 412(20) |
| C446T Ser132Phe | 3 | 405(2,3,4,5) 410(3,4) 412(21) |
| C447T Ser132Tyr | 3 | 405(2,3) 410(5) 412(20) |
| A448G Thr133Alab | 2 | 401(9) 410(4) |
| G451A Ala134Thr | 2 | 409(7) 413(22) |
| T457C Cys136Arg | 3 | 410(3) 412(21) 413(23) |
| T460C Ser137Pro* | 3 | 412(21) 413(22,23) 417(8) |
| T466C Ser139Pro* | 3 | 401(11) 414(17) 417(8) |
| G476A Arg142His* | 3 | 405(2) 410(3) 413(22) |
| T494C Leu148Ser | 2 | 401(9) 416(18) |
| A509G Lys153Arg* | 2 | 410(3) 414(22) |
| A512G Lys154Arg* | 3 | 401(9) 414(17,22) 415(13) |
| A518G Asn156Ser* | 3 | 409(9) 413(24) 415(13) |
| T520C Ser157Pro* | 3 | 405(2) 414(20) 416(18) |
| A535G Ser162Gly* | 2 | 413(22) 414(17) |
| G540T Lys163Asn* | 3 | 405(2,4) 410(3,4,5) 412(20) |
| T541C Ser164Pro* | 3 | 401(9) 412(20) 415(13) |
| T544C Tyr165His | 2 | 405(4) 414(17) |
| A551G Asn167Ser* | 2 | 405(4) 417(8) |
| A553G Asn168Asp* | 4 | 405(2,3,4) 406(18) 410(3,4,5) 412(20) |
| A554G Asn168Ser* | 2 | 412(20) 414(17) |
| A557G Lys169Arg* | 3 | 410(4) 412(20,21) 415(13) |
| A562G Lys171Glu | 2 | 400(23) 403(18) |
| A563G Lys171Arg | 2 | 403(18) 416(18) |
| T572C Leu174Pro | 3 | 405(4) 406(18) 410(5) |
| T587C Val179Ala | 2 | 401(9) 412(20) |
| A590G His180Arg^b^ | 3 | 401(9) 405(4) 414(19) |
| A607G Ser186Gly* | 5 | 400(19) 401(11) 405(2) 406(18) 412(21) |
| A614G Gln188Arg* | 2 | 406(18) 415(13) |
| A619G Ser190Gly* | 2 | 405(4) 414(19) |
| T623C Leu191Pro* | 2 | 414(19) 415(13) |
| G634A Ala195Thr* | 2 | 414(22) 417(8) |
| A638G His196Arg | 2 | 401(11) 413(24) |
| A644G Tyr198Cys | 2 | 413(22,23) 416(18) |
| G646T Val199Phe | 2 | 413(23) 416(18) |
| T647C Val199Ala | 4 | 405(2) 406(18) 410(3) 413(24) |
| C650T Ser200Leu | 2 | 403(18) 410(5) |
| T658C Ser203Pro* | 2 | 413(24) 414(22) |
| C662T Ser204Leu* | 2 | 405(3) 414(19) |
| A665G Lys205Arg* | 2 | 413(23) 414(21) |
| A671G Tyr207Cys | 2 | 405(4) 412(21) |
| T679C Phe210Leu | 2 | 409(7) 410(4) |
| T680C Phe210Ser | 2 | 400(23) 410(4) |
| A682G Thr211Ala | 3 | 405(3,4) 413(23) 414(22) |
| A691G Ile214Val | 2 | 405(5) 413(23) |
| A693G Ile214Met | 3 | 403(18) 413(23) 415(13) |
| G697A Ala216Thr | 2 | 401(11) 414(17) |
| G713A Arg221Lys* | 2 | 405(3) 406(18) |
| G715A Gly222Arg* | 2 | 405(4) 410(4) |
| A727G Arg226Gly | 2 | 405(5) 414(17) |
| A745G Thr232Ala | 2 | 405(2) 413(23) |
| A758G Gln236Arg* | 5 | 401(11) 405(4) 412(21) 415(13) 416(18) |
| A764G Asp238Gly | 5 | 400(19) 410(4) 412(21) 414(17,20,22) 415(13) |
| A766G Thr239Ala | 2 | 403(18) 412(21) |
| A769G Ile240Val | 2 | 405(2) 412(20) |
| A790G Asn247Asp | 2 | 405(3) 413(24) |
| A809G Tyr253Cys | 3 | 401(11) 416(18) 417(8) |
| G811A Ala254Thr | 2 | 410(3) 413(23) |
| T815C Phe255Ser | 2 | 405(5) 412(21) |
| C818T Ala256Val | 3 | 401(11) 412(20) 414(21) |
| A823G Asn258Asp | 2 | 405(5) 415(13) |
| A824G Asn258Ser | 3 | 405(2,3,4,5) 410(3,4,5) 412(20) |
| A826G Lys259Glu | 2 | 401(9) 412(21) |
| A827G Lys259Arg | 4 | 405(4) 406(18) 409(9) 410(5) |
| T838C Ser263Pro | 2 | 405(2) 412(20) |
| G844A Val265Ile | 3 | 405(2,3,4,5) 410(3,4,5) 412(20) |
| A850G Met267Val | 2 | 400(19,23) 414(17) |
| T853C Ser268Pro | 3 | 405(2) 409(9) 416(18) |
| A863G Gln271Arg | 4 | 400(19) 410(4) 413(24) 414(17,19) |
| T866C Val272Ala | 2 | 400(19) 410(4) |
| A869G His273Arg | 2 | 413(23) 416(18) |
| G875A Cys275Tyr | 2 | 400(19) 409(9) |
| T886C Cys279Arg | 3 | 400(19) 406(18) 413(22) |
| A890G Gln280Arg | 2 | 405(2,3) 412(20) |
| A892G Thr281Ala | 4 | 401(11) 409(9) 410(3) 412(21) |
| C896T Pro282Leu | 3 | 400(19) 410(4) 414(21) |
| C905T Ala285Val | 2 | 413(22) 416(18) |
| A913G Ser288Gly | 5 | 400(19) 405(4) 410(5) 412(21) 413(22) |
| A916G Asn289Asp | 2 | 412(21) 414(17) |
| T920C Leu290Pro | 3 | 400(19) 409(9) 410(3) |
| A929G Gln293Arg | 2 | 414(20) 416(18) |
| G934A Val295Ile | 2 | 400(19) 409(7) |

^§^ Signal peptide.

* Antigenic site.

^a^ Glycosylation site.

^b^ Receptor binding domain.

^c^ Day after initiation of the transmission experiment.
