# Supplementary material for: Evolution of an Eurasian Avian-like Influenza Virus in Naïve and Vaccinated Pigs
Source: PLoS Pathog. 2012 May 31;8(5):e1002730. doi: 10.1371/journal.ppat.1002730 (PMC3364949; doi:10.1371/journal.ppat.1002730)
Supplement: Table S5 — Stop codons detected in multiple pigs. (DOCX) [file ppat.1002730.s012.docx]

Table S5. Stop codons detected in multiple pigs.

| Study | Mutation | Amino acid position | Pig/s (days) | Direct contact? |
| --- | --- | --- | --- | --- |
| Naïve pigs | C361T | Gln104 | 104^a^(5),111^a^(7,8),106^a^(15) | yes |
|  | C487T | Arg146 | 115^b^(3),116^a^(6) | yes |
| Vaccinated pigs | G420A | Trp123 | 401^a^(7),412^a^(6,7) | no |

^a^ Pig infected through natural transmission.

^b^ Inoculated pig.
